# Evaluation of the patients with colorectal cancer undergoing emergent curative surgery

**DOI:** 10.1186/s40064-016-3725-9

**Published:** 2016-11-28

**Authors:** Fikri Kundes, Metin Kement, Kenan Cetin, Levent Kaptanoglu, Aytaç Kocaoglu, Mehmet Karahan, Serkan Fatih Yegen, Ali Emre Atici, Osman Civil, Mehmet Eser, Tebessum Cakir, Nejdet Bildik

**Affiliations:** Department of General Surgery, University of Health Sciences, Kartal Training and Research Hospital, Istanbul, Turkey

**Keywords:** Colon cancers, Rectal cancer, Colorectal cancers, Emergency surgery

## Abstract

**Background:**

The aim of our study is to evaluate perioperative and mid-term oncologic outcomes of the patients with colorectal cancer, who underwent emergent curative surgery.

**Methods:**

The study included all patients with colorectal cancer, who underwent surgery for curative intent between 1 January 2012 and 31 December 2014 in General Surgery Department of Kartal Training and Research Hospital. The patients were divided into two groups according to the type of admission (emergent or elective). The data of the patients were retrospectively collected with chart review. Demographic characteristics of the patients, ASA scores, emergent indications and surgical interventions, postoperative complications, pathological findings, oncological therapy, and follow-up findings were investigated.

**Results:**

Fifty-one and 209 patients were evaluated in both groups, respectively. Rate of right sided and sigmoid/recto-sigmoid tumors were significantly higher in emergent group. Ostomy rate, early morbidity, ICU need, transfusion, and mortality rates in emergent group were significantly higher than elective group. Average length of hospital stay in emergent group was also significantly longer in elective group (11.2 ± 3.2 vs. 8.4 ± 2.4 days). The patients in emergent group had a much lower survival rate than those in elective group.

**Conclusion:**

In our study, emergency presentation of colorectal cancer was found associated with increased morbidity, a longer length of stay, increased in-hospital mortality, advanced pathologic stage and worsened long term survival in even same stages.

## Background

Colorectal cancers are the third most frequent cancer in the west developed countries. They are also second cause of death in both men and women (World Cancer Research Fund and American Institute for Cancer Research 2007). Annually, it is estimated about 850,000 new cases and 500,000 deaths in worldwide (Ries et al. 1975–2005).

Although preventative measures and early detection programs, about 6–30% of patients with colorectal cancer admit with late complications, which requires emergent interventions. Those patients are usually at late stages and they are submitted to curative surgery in small proportion (Teixeira et al. 2015). Emergent surgical interventions for colorectal cancers are associated with a 15–20% of mortality and 40–50% morbidity, which are significantly higher than elective interventions (Tekkis et al. 2004). Also, bowel obstructions increase the risk of perforation, which is associated with increased rates of local recurrence.

The aim of our study is to evaluate perioperative and mid-term oncologic outcomes of the patients with colorectal cancer, who underwent emergent curative surgery.

## Methods

### Patients

The study included all patients with colorectal cancer, who underwent curative surgery for between 1 January 2012 and 31 December 2014 in General Surgery Department of Kartal Training and Research Hospital. The patients were divided into two groups according to the type of admission (emergent or elective). The data of the patients were retrospectively collected with chart review. Information about current status of patients was obtained by phone contact with the patients or their primary relatives. The study was approved by the Institutional Research Ethics Committee.

### Exclusion criteria

Exclusion criteria of our study were detection of peritoneal or distant metastasis, palliative interventions because of local advanced disease, R2 resection or pathologically positive surgical border (R1), insufficient lymph node dissection (below 12), the patients with rectal tumor who did not undergone resection for neo-adjuvant oncologic therapy, the patient undergoing only colostomy or stent placement, the patients with severe co-morbidities (ASA IV patients), the patients whose data could not be reached or did not want to anticipate to the study.

### Parameters

Demographic characteristics of the patients, ASA scores, emergent indications, diagnostic methods and surgical interventions, postoperative complications, pathological findings, oncological therapy, and follow-up findings were investigated.

### Statistical analysis

A retrospective analysis of a prospectively maintained database was undertaken. IBM SPSS Statistics 20 was used to perform the statistical computations. Categorical variables were compared using the Pearson Chi Square or Fisher’s exact test where appropriate. Continuous variables were compared using the 2 tailed Student t test or the Wilcoxon signed rank test where appropriate. Survival curves were computed using the Kaplan–Meier method and were compared between groups using log-rank test. A probability value of <0.05 was considered significant.

## Results

### Patients

**Fig. 1:**
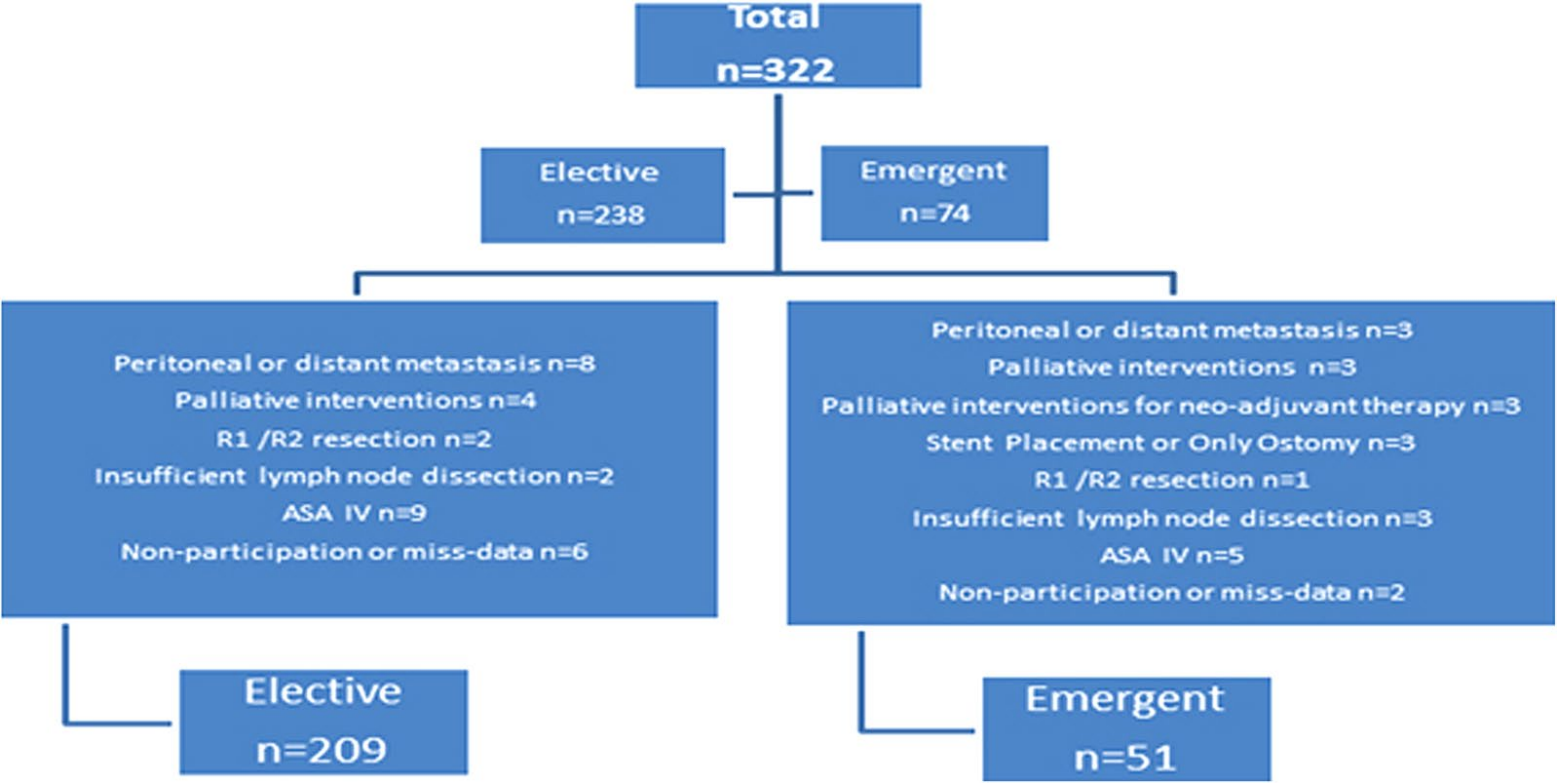
Twenty three (31.1%) and 31 (13%) patients were excluded for various reasons in emergent and elective groups, respectively

A total of 322 patients with colorectal cancer were undergone operation in our department in this period. Seventy-four of them (23%) were emergent cases. Twenty-three (31.1%) and 31 (13%) patients were excluded for various reasons in emergent and elective groups, respectively (Fig. 1). Finally, 51 (69.8%) and 209 (87.8%) patients were evaluated in both groups, respectively. Patients in emergent group were significantly older than those in elective group (66.4 ± 14.1 vs. 59 ± 11.3, P = 0.02). Gender was similar in two groups (62.8 vs. 58.9% male, P = 0.64) (Table 1).

**Table 1:** Demographic and clinical characteristics of the patients

Parameters	Emergent group	Elective group	P value
Mean age ± SD years	66.4 ± 14.1	59 ± 11.3	0.02
Gender (M, %)	32 (62.7)	122 (58.9)	0.64
ASA class III (%)	26 (51)	73 (35.3)	0.038
Ostomy (%)	22 (43.1)	24 (11.6)	<0.0001
Transfusion (%)	10 (19.6)	17 (8.2)	0.017
Surgical complication (%)	34 (66.7)	73 (35.3)	<0.0001
ICU (%)	27 (52.9)	52 (25.1)	<0.0001
Early mortality (%)	7 (13.7)	3 (5.8)	<0.0001
Hospital stay ± SD days	11.2 ± 3.2	8.4 ± 2.4	0.03
Pathological stage (III/II/I)	45/6/0 (88.2/11.8/0)	83/103/21 (40/49.8/10.2)	<0.0001

### Surgical indications

Emergency surgical indications were acute mechanical intestinal obstruction in 45 patients (88.2%), hollow organ perforation in 5 (9.8%) patients lower gastrointestinal bleeding in 1 (2%) in patient. In emergent group, primary diagnostic methods were colonoscopy in 28 (54.9%) patients, cross-sectional imaging (CT) in 14 (27.5%) patients and intraoperative exploration in 9 (17.6%) patients. Colonoscopy is primary diagnostic method in all the patients of elective group.

Tumor localization were the cecum in 10 (19.6%) patients, ascending colon in 4 (7.5%) patients, transverse colon in 4 (7.8%) patients, splenic flexure or descending colon in 5 (11.3%) patients, sigmoid colon or recto-sigmoid junction in 20 (39.2%) patients and rectum in 8 (15.7%) patients in emergent group. Comparison of tumor localizations between groups was presented in Table 2. Rate of right sided and sigmoid/recto-sigmoid tumors were significantly higher in emergent group. Furthermore, rate of rectal tumors was significantly lower in emergent group (Table 2).

**Table 2:** Comparisons of tumor localizations between groups

Localizations	Emergent group	Elective group	P
Cecum or ascending colon	14 (27.5)	29 (13.9)	*0.02*
Transverse colon	4 (7.9)	19 (9.1)	0.77
Descending colon	5 (9.8)	11 (5.2)	0.22
Sigmoid or recto-sigmoid colon	20 (39.7)	52 (24.9)	*0.03*
Rectum	8 (15.7)	98 (46.9)	*<0.0001*

Italic values indicate statistical significance

### Postoperative follow-up

In emergent group, postoperative surgical complications were developed in a total of 34 (66.7%) patients (surgical site infections in 24 (47%) patients, anastomotic leakage in 4 (10.4%) patients, stoma complications in 4 (12.9%) patients, prolonged ileus in 2 (3.9%) patients. Postoperative ICU was needed in 27 (52.9%) patients. Early postoperative mortality was developed 4 (7.8%) in those patients, the reasons of the mortality were septicemia secondary to anastomotic leakage in two patients and cardiopulmonary problems in two patients. Early morbidity, ICU need, transfusion, and mortality rates in emergent group were significantly higher than elective group. Average length of hospital stay in emergent group was also significantly longer in elective group (11.2 ± 3.2 vs. 8.4 ± 2.4 days) (Table 1).

The most frequently detected pathologic AJCC stage was III (n = 45, 88%) in emergent group. Rate of stage III was only 40.1% (n = 83) in elective group (P < 0.05). Pathological stages of the patients are shown in Table 3.

**Table 3:** Comparisons of pathological stages between groups

Stages	Emergent group	Elective group	P value
I	0 (0)	21 (10)	<0.0001
II a	1 (2)	72 (34.4)	
II b	3 (5.9)	20 (9.7)	
II c	3 (5.9)	11 (5.3)	
III a	0 (0)	25 (11)	
III b	21 (41.2)	37 (17.7)	
III c	23 (45.1)	21 (10)	

### Survival

**Fig. 2:**
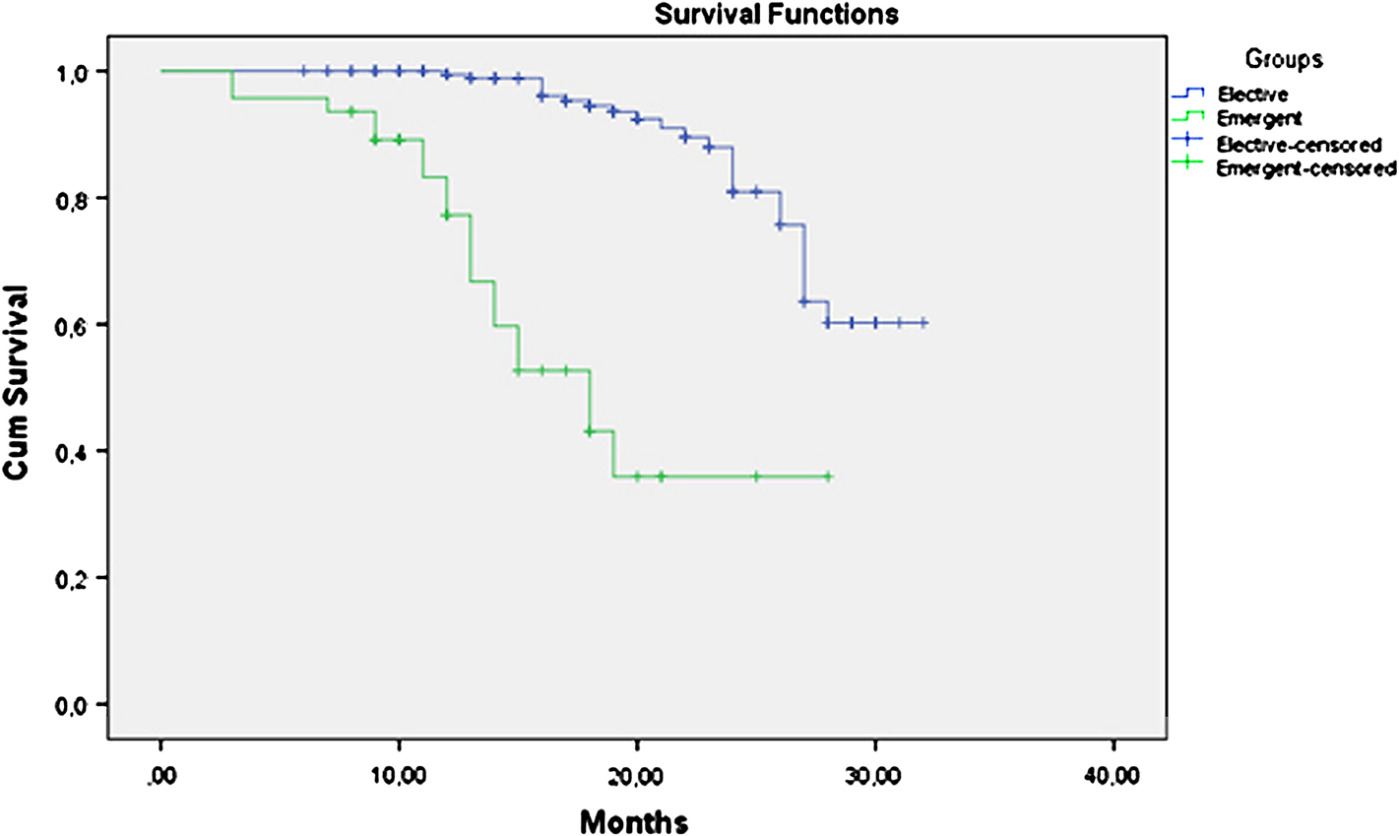
Estimated survival curves for two groups of patients in all stages

**Fig. 3:**
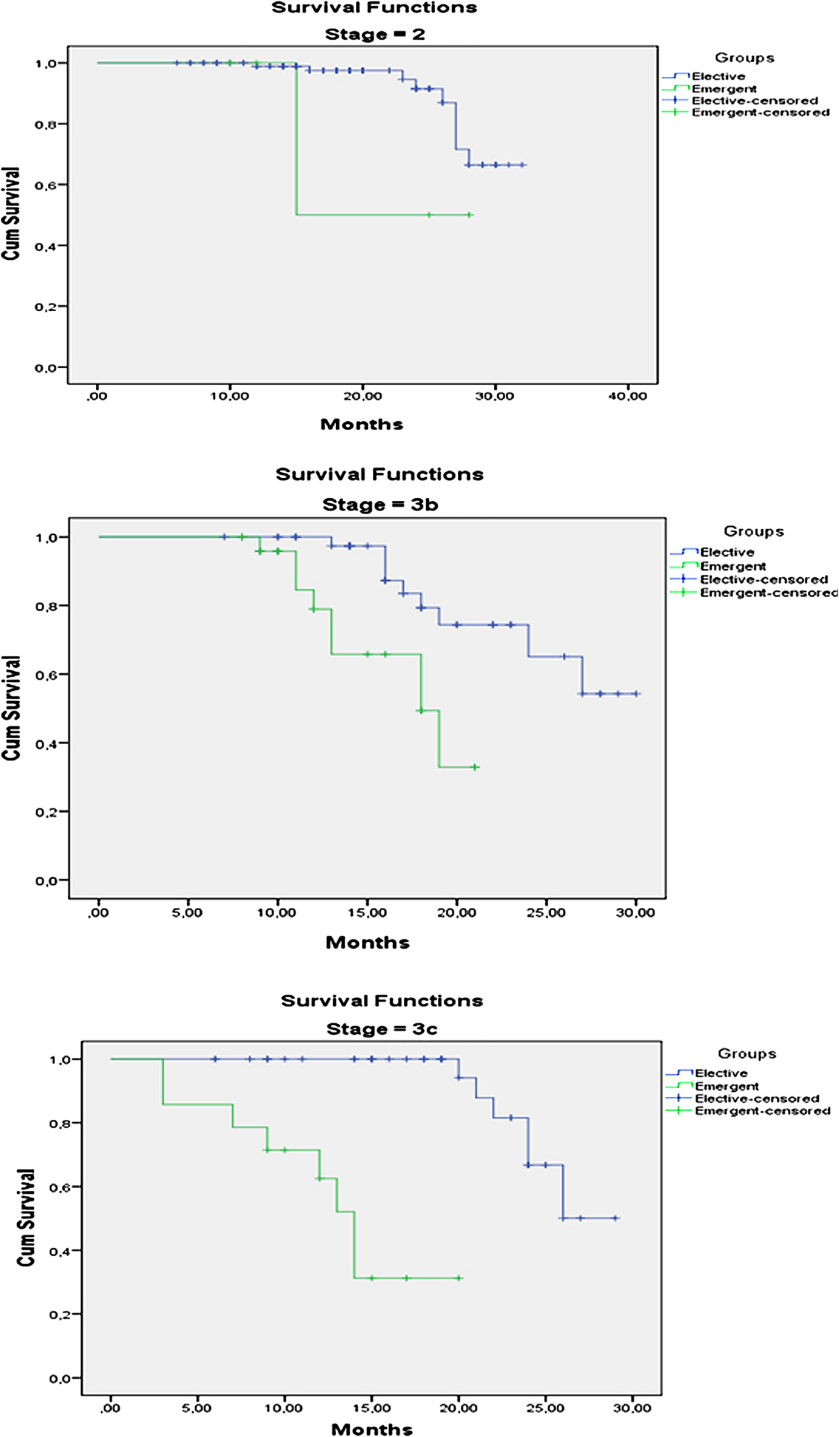
Estimated survival curves for two groups of patients in each stage (P < 0.05 for each comparisons)

Mean follow-up was 17.9 ± 6.7 months and 18.2 ± 7.3 in emergent and elective groups, respectively. In emergent group, 4 (7.8%) patients were lost in the early postoperative period (within 1 month), five patients (9.8%) died within 1 year after surgery. Fifteen (29.4%) patients died at postoperative 2 year. Twenty-two (43.1%) patients are still alive and 19 of them are disease-free. The patients in emergent group had a much lower survival rate than those in elective group with an estimated 2-years survival of 37 versus 82%, respectively (P < 0.001) (Table 4; Fig. 2). Furthermore, survival disadvantage for emergent group continued in same pathological stages (P < 0.05 for all comparisons) (Fig. 3).

**Table 4:** Means and medians for survival time in groups

Groups	Mean^a^				Median				Overall comparison		
	Estimate	SE	95% confidence interval		Estimate	SE	95% confidence interval		Log rank (Mantel Cox)		
			Lower bound	Upper bound			Lower bound	Upper bound	Chi square	*df*	Sig
Elective	28,787	551	27,707	29,866	–	–	–	–			
Emergent	18,599	1439	15,779	21,419	18,000	2303	13,486	22,514	69,269	1	0.000
Overall	27,381	590	26,224	28,537	–	–	–	–			

^a^Estimation is limited to the largest survival time if it is censored

## Discussion

Despite current screening programs, a large number of cases of patients with colorectal cancer present to the surgical clinic with emergent symptoms related to their malignancy. Countries with a national screening program, such as Australia, Germany, and Italy have reported emergency presentation rates of 6–19% contrary to rates of 22–34% in countries that do not have any program, such as Norway, Spain, and Ireland (Coco et al. 2005; Merkel et al. 2007; Wong et al. 2008; Biondo et al. 2005; Sjo et al. 2009; Bass et al. 2009). Our country does not have any screening program for colorectal cancers. Twenty-two percent of all colonic resections were carried out as emergency in our study. This rate is concordant with the literature.

The most common complication in colorectal cancer patients is bowel obstruction, followed by perforation and bleeding (Kronborg et al. 1975; Stower and Hard 1985; Carraro et al. 1998; Mandava et al. 1996). In our study, 88.2% of the patients had bowel obstruction. Nine point 8% of patients had perforation and only 2% of patients had bleeding. The most frequent tumor localization of our patients was sigmoid colon or recto-sigmoid junction (39.7%). The rate of right sided and transverse colon tumors were 27.5% in our study. Right sided and sigmoid/recto-sigmoid tumors were significantly higher in emergent group. Askari et al. from St Mark’s Hospital reported that the 263 patients who underwent emergency surgery, 37.3% had right-sided colonic cancers and they showed that right-sided tumors to be independently associated with undergoing emergency surgery (Askari et al. 2015).

In our study, 68.9% of emergent patients could undergo a potentially curative resection compared with 88.2% of elective patients. McArdle and Hole reported that 1603 (72.4%) of 2214 elective patients had a potentially curative resection compared with 632 (64.1%) of 986 patients who presented as an emergency (McArdle and Hole 2004). They suggested that the principles of oncologic resection for colorectal cancers operated on an emergency can be met, also achieving results related to the long term survival.

In a population-based study including 41,356 patients, Rabeneck et al. reported that advanced age, poverty, and lack of a family physician were associated with colorectal cancer emergencies (Rabeneck et al. 2006). Diggs et al. reported similar results in across-sectional study of 127,975 discharges of patients with colorectal cancer undergoing resection (Diggs et al. 2007). In our study, patients in emergent group were about 7 years older than those in elective group.

In our study, emergency presentation of colorectal cancer was found associated with increased morbidity, a longer length of stay, increased in-hospital mortality, advanced pathologic stage and worsened long term survival. The results of our study are quite concordant with the literature (Table 5). We also compared long term survival in the two groups according to stages. Emergency presentation was associated with worsened long term survival in even same stages. Coco et al. (2005) conducted a study of 787 patients where the 50 emergency patients were then matched for age, tumor location, stage, and comorbidities with 50 non-emergency case controls and found that the in hospital and long-term survival rates were the similar between the two groups. However, length of stay and complications in Coco’s study were higher in the emergency group despite being matched for pathologic stage.

**Table 5:** Comparisons of outcomes between emergent and elective presentation of colorectal cancer in current literature

Study	Country	Rate of emergency (%)	Hospital stay	Morbidity	30 day mortality	Long term survival	Pathological stage
Our study	Turkey	20	Longer	Higher	Higher	Lower	Advanced
Hwang (2012)	Canada	43	Longer	NS	NS	NS	Advanced
Bass et al. (2009)	Ireland	34	NR	NR	Higher	Lower	Advanced
Sjo et al. (2009)	Norway	25	NR	Higher	Higher	NR	Advanced
Biondo et al. (2005)	Spain	22	Longer	NR	Higher	Lower	Advanced
Wong et al. (2008)	Australia	19	NR	NR	Higher	Lower	Advanced
Merkel et al. (2007)	Germany	11	NR	NR	Higher	Lower	Advanced
Coco et al. (2005)	Italy	6	Longer	Higher	NS	NS	Not

Oliphant et al. examined postoperative mortality and longer-term survival by mode of presentation for patients with node-negative colorectal cancer undergoing curative surgery. They reported that 5-year relative survival was 91.8% after elective and 66.8% after emergency presentation (P  <  0.001) and the adjusted relative excess risk ratio for 5-year relative survival after emergency relative to elective presentation was 2.59 (95% CI 1.67–4.01; P  <  0.001) and 1.90 (95% CI 1.00–3.62; P  =  0.049) after exclusion of postoperative deaths (Oliphant et al. 2014).

As our study indicated, emergency surgery for colorectal cancers is generally associated with a much higher morbidity and mortality rates, when compared to elective surgery. These undesirable outcomes are mostly attributed to the advanced age, frequent co-morbidities, malnutrition and advanced stage of disease (Runkel et al. 1998). Although outside the scope of our study, the use of a bridge to definitive surgery by using either ostomy or stent placement in left sided tumors may be alternative to emergent resection. Thus, a time can be created to optimize the patients’ condition, let the dilated bowel restore and perform further tumor staging. Encouraged by good outcomes from multiple retrospective studies on stent placement this appeared to be a very promising treatment option. Nevertheless, the early closure of numerous randomized controlled studies investigating the role of stents in colorectal cancers because of stent related complications have caused cautiousness towards this approach. Particularly stent-related perforation is a feared complication. In the past years, several risk factors for stent-related colonic perforation were identified (van Hooft et al. 2008; Cheung et al. 2009; Pirlet et al. 2011). In addition to a possibly high complication rate, questions have been raised about the oncologic long-term results following stent placement, since it is thought that tumor manipulation by stent placement possibly leads to micro-perforations and tumor spill. However, only a few studies have reported on long-term outcomes (Erichsen et al. 2015; Sloothaak et al. 2014), a recent systematic review recommended stent placement in only palliative and unfit patients, which is in accordance with the most recent ESGE Guideline (van Hooft et al. 2014; Zhoa et al. 2014; Frago et al. 2014). ESGE guideline states that colonic self-expandable metal stent (SEMS) placement as a bridge to elective surgery is not recommended as a standard treatment of symptomatic left-sided malignant colonic obstruction and for patients with potentially curable but obstructing left-sided colonic cancer, stent placement may be considered as an alternative to emergency surgery in those who have an increased risk of postoperative mortality, i.e. ASA Status ≥III and/or age >70 years. Therefore, stent placement as bridge to surgery should not be performed in relatively fit patient patients with potentially curable cancer, colostomy creation is more appropriate option for bridging to surgery in with potentially curable patients and patients with risk factors for perforation. A recent systematic review, which provided an overview of all available literature on primary resection versus colostomy creation as bridge to surgery in patients with acute left sided colorectal tumors, concluded that a diverting colostomy as bridge to surgery is a safe and valid alternative for primary resection (Amelung et al. 2015).

This study has some limitations which have to be pointed out. First of all, it was a single center retrospective study with relatively small cohort. Furthermore, the follow-up period was quite short and did not include 5 years data. The heterogeneity of the patients (with colon and rectum cancer) was another limitation of our study.

## Conclusion

In our study, emergency presentation of colorectal cancer was found associated with increased morbidity, a longer length of stay, increased in-hospital mortality, advanced pathologic stage and worsened long term survival in even same stages.

## Abbreviations

ASA: The American Society of Anesthesiologists
ICU: intensive care unit
CT: computed tomography
ESGE: European Society of Gastrointestinal Endoscopy
SEMS: self-expandable metal stent
